# Potentiometric Electronic Tongue to Resolve Mixtures of Sulfide and Perchlorate Anions

**DOI:** 10.3390/s110303214

**Published:** 2011-03-16

**Authors:** Deivy Wilson, Mohammed N. Abbas, Abdel Latief A. Radwan, Manel del Valle

**Affiliations:** 1 Sensors and Biosensors Group, Department of Chemistry, Universitat Autònoma de Barcelona, 08193 Bellaterra, Spain; E-Mail: dwilson25@gmail.com; 2 Analytical Laboratory, National Research Centre, Dokki, Cairo, Egypt; E-Mails: mnabbas@menanet.net (M.N.A.); celotfy@yahoo.com (A.L.A.R.)

**Keywords:** electronic tongue, ion-selective electrode, artificial neural network, sulfide, perchlorate, phtalocyanine ionophores

## Abstract

This work describes the use of an array of potentiometric sensors and an artificial neural network response model to determine perchlorate and sulfide ions in polluted waters, by what is known as an electronic tongue. Sensors used have been all-solid-state PVC membrane selective electrodes, where their ionophores were different metal-phtalocyanine complexes with specific and anion generic responses. The study case illustrates the potential use of electronic tongues in the quantification of mixtures when interfering effects need to be counterbalanced: relative errors in determination of individual ions can be decreased typically from 25% to less than 5%, if compared to the use of a single proposed ion-selective electrode.

## 1. Introduction

The monitoring of sulfide and perchlorate is required in a variety of environmental and industrial situations, as both ions form toxic compounds. The toxicity of sulfide is well known, especially when released as the hydrogen sulfide (H_2_S) form; this gas at high concentrations can cause permanent brain damage and even death due to its neurotoxic effects [1].

Perchlorate may be found at high concentration levels (higher than 1,000 ppm) in surface and ground waters [2], and has also been found in food products, soil, milk, fertilizers, plants and in human urine. One of its major sources of pollution comes from its use in solid propellants, explosives and other industrial uses. Perchlorate may interact with the production of thyroid hormones and its presence has been related to the occurrence of thyroid cancer.

The sulfide and perchlorate determination can be carried out by a variety of analytical techniques, some of them classical such as titrimetric and gravimetric, or employing instrumental techniques, such as chromatography, atomic absorption, electrochemistry and combinations thereof [3]. However, most of these methods are relatively expensive in terms of analysis time or the need for sophisticated instruments.

In recent years, the use of Ion Selective Electrodes (ISEs) has become a good alternative [4,5] for water monitoring applications; today there are many ISEs commercially available, but these can present limitations with respect to selectivity and utilizable pH. Another feature that may limit the utility of selective electrodes is the short lifetime of their sensing membranes due to exudation of the ionophore from the polymer matrix. A recent trend to prevent the occurrence of leaching phenomena reported in the literature is the immobilization of the ionophore on the matrix [6]. Other authors also report this method as a means to improve the detection limit [7].

At present, in the sensor community, the use of the term ¨electronic tongue¨ is not novel. According to the recent IUPAC definition [8], an electronic tongue (ET) is “a multisensor system, which consists of a number of low-selective sensors and uses advanced mathematical procedures for signal processing based on Pattern Recognition and/or Multivariate data analysis—Artificial Neural Networks (ANNs), Principal Component Analysis (PCA), *etc*”. Therefore, the electronic tongue is an analytical system devised for liquid analysis and formed by a sensor array that generates multidimensional information, plus a chemometric processing tool which extracts meaning from these complex data [9]. There are many applications found in the literature for qualitative [10–12] and quantitative analysis [13–15], the first type being the most favored if the amount of publications is concerned. Electronic tongues and electronic noses, two bioinspired sensor analysis systems—the latter is equivalent but used for gas analysis—are consolidated trends in the sensor’s field, for the number of works already published, and for the number of different laboratories working with them [16,17].

One important concept in the electronic tongue is the cross-response issue. The sensor array, part of the ET, needs to use sensors with reduced selectivity and mixed sensitivity, in order to generate the multidimensionality required to develop the application. Although other types of sensors may be involved, many of the ETs described in the literature use arrays of ISEs, and in the case studied here, we selected to study the ISE-based multidetermination of perchlorate and sulfide anion mixture. This kind of quantitative applications are specially demanded for pollution monitoring [13], but similar anion determinations have been used for characterizing underground waters [18] and also for qualitative analysis of beverages [19]. In the choice of potentiometric sensors for anions, there are two clear options: to use ISEs based on quaternary ammonium ion carriers [20], which normally present a certain response preference for the lipophilic anions (Hofmeister series), or to use new families of carriers in order to deviate from that, and generate a richer cross-response in the array. The choice in this study was to mix both; in particular, we have employed different metallophtalocyanines [21–23] to obtain an anti-Hofmeister response pattern. In order to obtain sensors with better stability and lifetime characteristics, some of the metallophtalocyanine ionophores used have been linked by covalent bond to acrylic polymers, in order to favor their permanent attachment to the sensing membrane [6]. Durable perchlorate and sulfide solid-contact ISEs, based on ionophores Cobalt-Phthalocyanine and Cerium(IV) [*N*′-acetyl-2-(benzothiazol-2yl)-3-(3-chloro-5-methyl-4*H*-pyrazol-4-yl)acrylohydrazide] complex, respectively, have been covalently attached to polyacrylamide (PAA) and used in an electronic tongue for the simultaneous determination of sulfide and perchlorate in pollution studies.

## 2. Experimental Section

### 2.1. Reagents

All chemicals used were of reagent grade and doubly distilled water was used throughout all preparations. Plasticizers *o*-nitrophenyloctyl ether (*o*-NPOE), dioctyl phthalate (DOP), dibutyl phthalate (DBP), dibutyl sebacate (DBS), [*N*′-acetyl-2-(benzothiazol-2yl)-3-(3-chloro-5-methyl-4*H*-pyrazol-4-yl)acrylohydrazide] (ABPAH), polyacrylamide (PAA), gallium phthalocyanine (Ga-Pc), zinc phthacyanine (Zn-Pc), cobalt phthalocyanine (Co-Pc) and high molecular weight PVC were supplied by Aldrich. The oleic acid (OA), cetyltrimethylammonium bromide (CTMAB), tetradodecylamonium bromide (T12A) and tetrahydrofuran (THF) were obtained from Fluka. Sodium dodecylbenzenesulfonate (SDBS) was obtained from Carlo Erba. Sodium sulfide, sodium perchlorate, phosphoric acid, sulphuric acid, dimethylformamide (DMF), phosphorus pentoxide and cerium (IV) sulphate were purchased from Merck or Aldrich and were of the highest purity available. All the anions working solutions were freshly prepared by accurate dilution from their 0.1 M stock solutions renewed periodically.

### 2.2. Apparatus

The emf measurements were performed with a laboratory made data acquisition system consisting of 32 input channels made with differential instrumentation amplifiers (INA116, Burr-Brown, USA) that adapted the impedance for each sensor. Emf measurements were performed against a double junction Ag/AgCl reference electrode (Thermo Orion 90-02-00). Each channel was noise-shielded with its signal guard. The output of each amplified channel was filtered with a second order low pass active filter centered at a 2 Hz frequency and connected to an Advantech PC-Lab 813 A/D conversion card installed in a PC computer.

### 2.3. Preparation of the Cerium(IV)-ABPAH Complex

An amount of 0.01 mole of Ce(SO_4_)_2_·H_2_O dissolved in 0.01 M H_2_SO_4_ was added dropwise to ethanolic solution of 0.01 mole of the ABPAH, with continuous stirring whereby pale yellow, brownish–yellow, and yellow precipitates were obtained, respectively. The precipitate was washed thoroughly with water and ethanol, and then allowed to dry in air at ambient temperature. The structure of this ionophore was confirmed by UV-VIS, IR and elemental analysis.

### 2.4. Synthesis of the Covalently Attached Ionophores

An appropriate amount of the ionophore (cerium complex or Co-Pc) was added to 900 mg of PAA in 50 mL of DMF in the presence of P_2_O_5_ and H_3_PO_4_ as dehydrating agent, then the mixture was purged with nitrogen gas for about 6 hours, the mixture was refluxed for about 24 hours at 140 °C, the precipitate was filtered and washed with water, ethanol, then let dry at ambient temperature to obtain the corresponding product. The product was investigated by IR analysis.

### 2.5. Sensor Array

The sensor array used was formed by five potentiometric sensors (all-solid-state ISEs), which were constructed as follows: the appropriate amounts of the specified ionophore, PVC, additive and the plasticizer were placed in a 10 mL vial and mixed thoroughly. Then the mixture was dissolved in 5 mL THF under magnetic stirring until the prepared membrane cocktail became clear. A clean and dry carbon rod of 5 cm length and 5 mm diameter was dipped to about 1 cm depth into the membrane cocktail for 2 s, and then lifted out of the solution to evaporate the THF, leaving the polymeric membrane layer coating the carbon rod. That operation was repeated for 12–17 times to give a proper membrane thickness. The rod was fitted into a plastic body and connected with the membrane-free end to the potentiometer using a copper wire. The constructed ISEs (see Table 1) were conditioned for 24 hours by soaking in 1.0 × 10^−3^ mol L^−1^ solution of its primary ion prior to use. The Zn, Ga and Co metallophtalocyanine ionophores were prepared according to the literature [23]. Apart, a quaternary ammonium membrane was also included in the array, in order to provide a different selectivity pattern; in this case the ionophore used was the ion pair tetradodecyl ammonium dodecylbenzenesulphonate (T12A-SDBS) [24], capable of responding to the different anions involved in this study case.

### 2.6. Procedure

The performance characteristics of the used sensors: detection limit, selectivity coefficient, slope, were determined according to IUPAC methodology. The activity coefficients of ions in solution were calculated according to the Debye-Hückel formalism [25].

To build the response model, a number of the two anion mixtures were sequentially prepared from cumulative additions of standard solutions of increasing concentrations of one or two considered ions. To this end, microvolumes of the standards were added to the calibration vessel with the aid of variable-volume micropipettes. The solutions used contained Na_2_S and NaClO_4_, whether individually or in binary combinations; these standards were prepared from solutions of concentration 10^−4^, 10^−3^, 10^−2^, 10^−1^ and 1 M of each ion. The more concentrated solution was prepared by direct weighing of the salts and all other by sequential dilution. All measurements were carried out without any background or buffer solution. For the simultaneous mixture determination, a two-ion response model was built, feeding the responses from the sensor array to an artificial neural network structure.

### 2.7. Software

The readings were acquired by using custom software developed by our group and written in Microsoft QuickBasic Version 4.5. Neural network processing was developed with MATLAB 6.0 (Mathworks, Natick, MA, USA), using its Neural Network Toolbox (v. 3.0). Sigma Plot 2000 (SPSS Inc., Chicago, IL, USA) was used in graphic representation of data.

## 3. Results and Discussion

### 3.1. ISE Responses

A preliminary characterization was made to the above prepared ISEs before being used in the electronic tongue. Measurements were carried out in order to build the response model employing ANN and to perform the multidetermination application.

**Figure 1.**
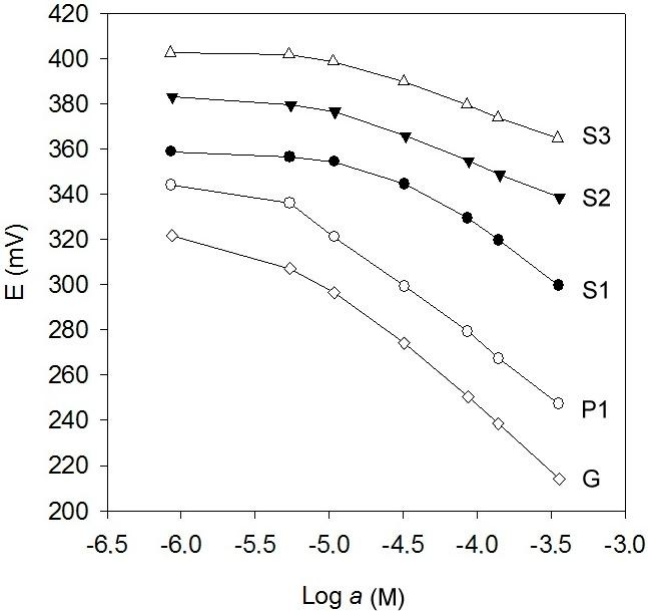
Response of the ISEs forming the sensor array towards its primary ion: ISEs to perchlorate P1 (○); to sulfide S1 (•), S2 (▾) and S3 (Δ); and generic ISE for sulfide (◊).

Figure 1 shows the response of each prepared sensor type to their respective primary ions; calibration curves represented in the figure were obtained by adding a background saline solution (0.05 M lithium acetate), to adjust the pH and ionic strength of solutions, reducing the effect of the change in pH by hydrolysis of sulfide ion. Table 2 shows the main performance characteristics obtained. The responses of ISEs (P1, S1, S2, S3) are close to Nernst values, while the sensitivity to S^2−^ of the generic sensor is super-Nernstian probably due to a mixed response to S^2−^ and HS^−^. Apart, the detection limits are below 10^−5^ mol L^−1^; this concentration is taken as a lower limit on the working range. Particularly, the perchlorate ISE P1 displayed remarkable response characteristics, closely to Nernstian ideality [26]. The reproducibility of response for all proposed sensors showed to figure between 0.96 and 0.98% RSD of slope between successive calibrations of the primary ion on five consecutive days.

One of the most important premises for constructing an ET is the cross-selectivity of the sensor array [8,27]. In our case, this was verified by calibrations in presence of primary and interfering ion (Mixed Solution Method), which permitted to calculate the potentiometric selectivity coefficients according to the Nikolsky-Eisenmann Expression (1):

(1) $$E_{i}=K_{i}+s_{i}\cdot \log\left[a_{i}\;+\;k_{i,j}^{\mathrm{pot}}\;\cdot \;\;{\left(a_{j}\right)}^{z_{i}/z_{j}}\right]$$

The results from these evaluations are summarized on Table 2. According to the obtained selectivity coefficients, $k_{i,j}^{\mathrm{pot}}$, we can infer that electrodes P1 based on (Co-PC-PAA) and S1 based on (Zn-PC) have clear cross-response to both ions, as it also happens with the generic ISE; similarly, ISEs S2 and S3 show marked selectivity to sulfide, as they show a difference in response of three orders of magnitude. Sensors Ce-ABPAH-PAA for S^2−^ and Co-Pc-PAA for ClO_4_^−^ in which the ionophore is covalently bonded to the polymer matrix, showed marked stability in the slope and detection limit for 16 weeks of measurement. Lifetime of the remaining sensors was slightly poorer, 8–10 weeks.

### 3.2. Building of the ANN Response Model

In our studies, the preferred chemometric tool for the advanced processing of data has been the Artificial Neural Network (ANN). These are known to be powerful non-linear modelers, applicable for quantitative and also qualitative applications [28]. In this sense, our approach is doubly biomimetic; firstly, the use of groups of sensors with cross-response is the sensing scheme in the taste buds of animals, and secondly, ANNs are parallel information processing tools inspired in the animal nervous system, whose maximum expression is the human brain. Consequently, ANNs were used to model the combined response of the two ions mixture from the readings of the sensor array.

**Figure 2.**
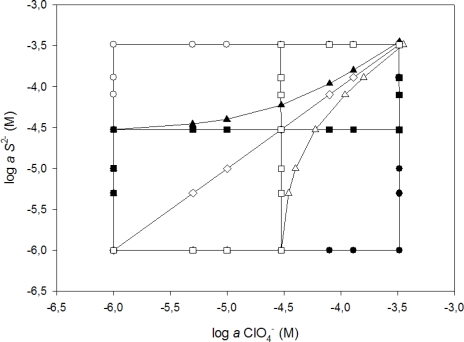
Visualization of the standards generated for the training of the electronic tongue, following the seven sequences of additions: 1(○), 2 (•), 3 (□), 4 (▪), 5 (▵), 6 (▴), 7 (⋄).

For the building of the response model, specific experimentation was designed to obtain the information needed. The starting data consisted of 79 samples (mixtures of the two considered ions S^2−^ and ClO_4_^−^) covering the two species concentration range, and was split into two subsets: a training subset containing 59 samples, used for building the response model, and a test subset containing 20 samples, used for evaluating the model predictive ability. The samples were generated by seven different sequences of microvolume additions [29] of two concentration levels of ClO_4_^−^ and S^2−^ standards, alone or combined, which allowed generating the experimental design in Figure 2. Finally, the response model corresponded to the symmetric concentration range of 5.0 × 10^−6^ to 3.3 × 10^−4^ M for the two target ions (ClO_4_^−^ and S^2−^). To divide the data subsets it is very important to do it randomly [30] and to avoid that samples corresponding to maxima or minima may be in the testing subset, in this way the need of any extrapolation is eliminated [31]. Each subset contains two kinds of information that interrelates: the first type is formed by the responses of the sensor array (patterns); the second is the sought information in correspondence (targets), which in a quantitative application case are the concentration values of the analytes. This training subset must be large enough, cover adequately the original space and contain sufficient variability to yield a proper modeling of the response. The random distribution of these points, plus extra precautions to avoid overfitting are key issues in order to obtain confidently valid response models [32].

Selecting the topology of an ANN is the first task in developing a numeric model with ANNs because of the difficulty to predict an optimum configuration in advance. The ANN structure for the best modeling of a sensor array is obtained by a trial and error procedure. The optimization process includes verifying a combination of training strategies, their associated parameters, the dimension of the hidden layer and the transfer functions to be used in the hidden and output layers. These characteristics will define the specific configuration leading to the best modeling ability.

Certain characteristics of the ANN configuration were initially fixed. These included the number of input neurons, which was five (the five sensors from the array); the number of output neurons, which was two (the two modeled analytes); the transfer function of the output layer, which was linear (purelin) and a single hidden layer of neurons. These selections are based on previous experience with electronic tongues using potentiometric sensors [27,30,33]. The learning strategy used was Bayesian Regularization and employed, for its internal parameters, a learning rate of 0.1 and a momentum of 0.4, selected from preliminary tests. The modeling capability of the ANN was examined in terms of the root mean squared error (RMSE) of the concentrations sought, and in the comparison graphs of predicted *vs.* expected concentrations for the two ions.

When compared with others, the strategy selected for the learning process (Bayesian Regularization) provided better RMSE value, greater consistency between the predicted and obtained values for the training, a higher significance for the external test set and, besides, an internal validation subset of samples was not necessary given it avoids overfitting by other means [34]. In our case, parameters such as the number of neurons in the hidden layer, and the transfer function used in the hidden layer were varied systematically in order to get the best final performance.

**Figure 3.**
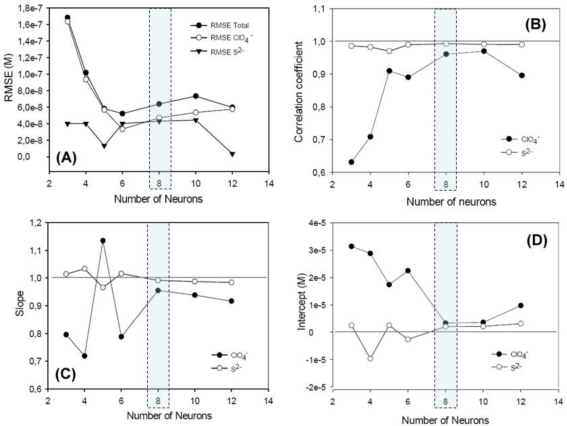
Selection of the optimal number of neurons in the hidden layer for the ANN model using tansig and purelin transfer functions for hidden and output layer respectively. **(A)** RMSE values, **(B)** correlation coefficients, **(C)** obtained slopes and **(D)** intercepts for the comparison regression between obtained *vs.* expected concentrations.

Figure 3 shows a summary of the results obtained during the optimization of ANN features, the number of neurons in the hidden layer is selected as eight and the transfer functions were tansig (hyperbolic tangent sigmoid) and purelin (linear) for hidden and output layer respectively. The factors considered for the selection were an accuracy of model fit, calculated with smaller RMSE (root mean squared error), and correct prediction abilities, as shown in the obtained *vs.* expected comparison graphs for the two ions, where their slope, intercept and correlation comparison parameters should approximate 1, 0 and 1 for best performance.

**Figure 4.**
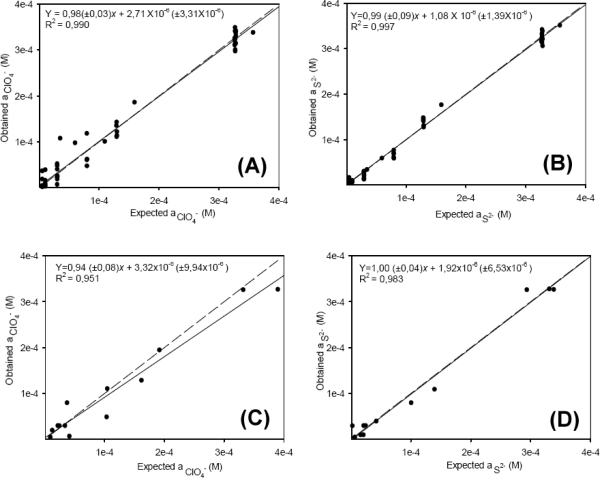
Modeling performance achieved for the optimized ANN with samples from the training test (A,B) and the external test set: (C,D): on the left, perchlorate ion; on the right, sulfide ion. The dashed line corresponds to ideality, and the solid line is the regression of the comparison data. Uncertainty intervals calculated at the 95% confidence level.

Figure 4 illustrates the behavior of the modeling system, and the correlation between the obtained (y) and expected (x) values for the training and external test subsets of the two individual ions. As can be seen the model prediction is very good for both ions, the accuracy of the obtained response is adequate, with unity slope and zero intercept (all confidence intervals were calculated at the 95% confidence level). In Figure 4(C) (perchlorate, external test subset) a greater dispersion is observed for this ion; the presence of a single ClO_4_^−^-ISE in the sensor array maybe the cause of these results. In order to check whether the results corresponded to a local area or a global minimum of the system, the ANN was trained five times, the weight values for the neurons being reset at random each time in order to estimate the precision of the model.

### 3.3. Application

**Figure 5.**
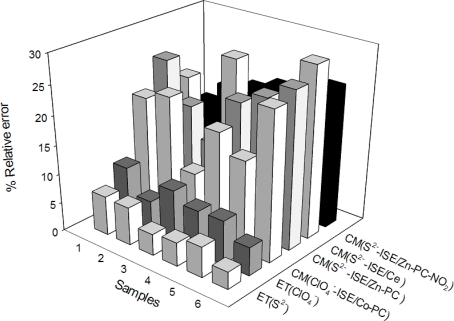
Absolute errors (%) obtained for the simultaneous determination of sulfide and perchlorate in synthetic samples by the proposed electronic tongue and by use of standard potentiometry with each individual ISE in the array.

To evaluate the performance of the sensor array, six wastewater samples with initial concentrations of 0.3 × 10^−6^ to 3.4 × 10^−5^ M of S^2−^ and ClO_4_^−^ were spiked with standard additions of the two ions 0.01 M to obtain a maximum concentration of 5.0 × 10^−4^ M for both ions.. Their concentrations were predicted employing the ANN response model built previously. Figure 5 shows the relative error obtained for the wastewater samples. The two first rows ET (S^2−^) and ET (ClO_4_^−^) are the results obtained by the optimized ET, the other ones are the results of the conventional method: by interpolation in the calibration curve for each individual electrode. As can be seen, the error values obtained in the determination of the two target ions with the ET are in the order of 5% and always lower than the result obtained with conventional method using the equivalent single electrode. The determination of perchlorate concentration was less precise than the sulfide determination and the reason could be the same as above: the array contained a single perchlorate ISE.

In order to judge the goodness of the results obtained employing the proposed ET methodology, a Student’s paired samples t-test was performed. The calculated t statistics are: t = 0.11 for the ClO_4_^−^ ion and t = 1.52 for the S^2−^ ion. In both cases, the calculated t statistics were clearly below the tabulated critical value of t* = 2.57 (5 degrees of freedom and 95% confidence level), demonstrating that there were no significant differences between results obtained with the sensor array and the nominal concentration of the synthetic samples.

## 4. Conclusions

An electronic tongue with potentiometric sensors for the simultaneous determination of perchlorate and sulfide in synthetic samples and wastewaters was developed. The sensor array was formed by specific ISEs formulated with metallophtalocyanines, which give diverse response patterns to the anions considered. Some of the membranes were prepared with its ionophores covalent linked to a polymeric support allowing for a long-living sensor with improved performance and selectivity. The combined response was modeled with an ANN, showing better performance characteristics than when compared with individual sensors used in standard way, which permits to recommend the electronic tongue for studying polluting episodes of this nature. The followed procedure did not require any pretreatment of the sample, in a simpler manipulative procedure.

## Figures and Tables

**Table 1.** Formulation of the ion-selective membranes employed in the construction of the potentiometric sensor array.

**Membrane**	**Primary ion**	**Ionophore (wt.%)**		**Plasticizer (wt.%)**		**PVC (wt.%)**	**Additive (wt.%)**	
				***o*-NPOE**	**DOP**		**OA**	**CTMAB**
P1	ClO_4_^−^	Co-Pc-PAA	9.30	-	57.80	27.77	2.60	2.60
S1	S^2−^	Zn–Pc	3.22	64.52	—	32.26	—	—
S2	S^2−^	Ce-ABPAH-PAA	4.76	63.49	—	31.75	—	—
S3	S^2−^	Ga-Pc	4.16	—	64.59	31.25	—	—
G	Generic	T12A –SDBS	12.50	62.50	—	25.00	—	—

**Table 2.** Response characteristics of the ISEs forming the sensor array.

**ISEs**	**Slope, *s_i_* (mV/decade)**	**LD (mol·L^−1^)**	${\log k}_{i,j}^{\mathrm{pot}}$	
			**ClO_4_^−^ interfering ion**	**S^2^^−^ interfering ion**
P1	−57.7	5.2.10^−6^	—	−1.96
S1	−33.3	1.3.10^−5^	−2.68	—
S2	−30.9	9.8.10^−6^	−3.23	—
S3	−28.9	9.9.10^−6^	−3.75	—
G	−44.3 ^a^	8.3.10^−6^	1.13	—
	−60.1 ^b^	7.5.10^−6^	—	−1.25

a Response to S^2−^ ion;

b Response to ClO_4_^−^ ion.
